# The Role of Prospective Memory in Predicting Neuropsychological Functioning in Mild Cognitive Impairment and Alzheimer’s Disease

**DOI:** 10.1002/alz.088562

**Published:** 2025-01-03

**Authors:** Jordan Kozuki, Andrea Collins, Fabiola Amaya Reyes, Summer Herrera, Keely Cruz‐Lopez, Ellen Woo

**Affiliations:** ^1^ California State University, Fresno, Fresno, CA USA; ^2^ University of California, San Francisco ‐ Fresno, Fresno, CA USA

## Abstract

**Background:**

Prospective memory (PM), the ability to execute an intended action in the future (e.g., taking medications), is impaired in Alzheimer’s disease (AD). This study examines the utility of subjective and objective measures of PM in predicting neuropsychological functioning across five cognitive domains in healthy aging, mild cognitive impairment (MCI), and AD.

**Method:**

Participants included 58 healthy older controls, 65 persons with MCI, and 18 individuals with AD. Subjective reports of PM included both participant and informant ratings on the Prospective‐Retrospective Memory Questionnaire (PRMQ). Additionally, an objective PM task was completed throughout a neuropsychological test battery. Simple objective PM was measured by asking participants to remember to execute the single action of requesting a pill after each neuropsychological test performed. Complex objective PM was measured by asking participants to request a specific number of pills depending on whether a memory or non‐memory test was completed. Composite neuropsychological test scores were used to assess the domains of language, memory, executive functioning, visuospatial skills, and attention.

**Result:**

Regression analyses revealed that in MCI, informant reports of PM predicted executive functioning. In healthy aging and MCI, both types of objective PM predicted memory performance. PM did not predict language, visuospatial skills, or attention in any group.

**Conclusion:**

Among healthy older adults and those with MCI, objective measurements of prospective memory predict episodic memory outcomes. When participants begin to exhibit cognitive decline, as seen in MCI, informant reports are additionally beneficial and predict executive functioning. This research supports previous findings that prospective memory is multifactorial and involves both memory and executive functioning. Overall, this research also suggests that the assessment of prospective memory has utility for understanding the early decline of cognitive domains traditionally evaluated in MCI.

